# Public Interest in COVID-19 Therapeutics for High-Risk Populations During the Omicron Era: A Google Trends Analysis

**DOI:** 10.7759/cureus.32684

**Published:** 2022-12-19

**Authors:** Huseyin Berk Degirmenci, Jinseo Oh, Alison M Bays, Jenna L Thomason, Jean W Liew

**Affiliations:** 1 Department of Medicine, St. Elizabeth's Medical Center, Tufts University School of Medicine, Boston, USA; 2 Section of Rheumatology, Department of Medicine, Boston University School of Medicine, Boston, USA; 3 Division of Rheumatology, Department of Medicine, University of Washington, Seattle, USA

**Keywords:** public health, pre-exposure prophylaxis, google trends, covid-19, evusheld

## Abstract

Therapies for COVID-19 prevention or treatment continue to play a significant role for individuals who are not able to mount an adequate immune response after COVID-19 vaccination and/or in patients who are at high-risk for severe outcomes of COVID-19 infection. As these modalities have become more available, it is important to assess the public’s interest in these agents to ensure both patients and physicians are aware of the therapeutics available to them. Google Trends is a freely available tool that researchers can use for monitoring public interest by analyzing trends in search queries during disease outbreaks. In this descriptive study, we used Google Trends to investigate the public interest in two COVID-19 therapeutics which received Food and Drug Administration (FDA) emergency use authorization in December 2021: Paxlovid, an antiviral medication used for COVID-19 treatment, and Evusheld, a combination of two monoclonal antibodies against COVID-19 used for COVID-19 prophylaxis. We analyzed search queries in the first half of 2022. Our analysis included search queries that include ''Paxlovid'', ''Evusheld'', ''COVID treatment'' and ''COVID prophylaxis'' at the national and state levels in the US. We found that while the number of COVID-19 cases rose during the period of interest, Evusheld searches remained stagnant despite a concurrent increase in Paxlovid searches. These findings potentially represent low public interest or awareness about Evusheld, which can be addressed through public health initiatives to ensure improved distribution.

## Introduction

Coronavirus disease 2019 (COVID-19) was declared a global pandemic by the World Health Organization (WHO) on March 11, 2020 [1]. Since then, multiple interventions have been developed for both treatment and prevention, such as vaccinations, targeted antiviral medications, and monoclonal antibodies. 

COVID-19 vaccines provide strong protection against severe diseases [2]. However, humoral responses following the vaccine for severe acute respiratory syndrome coronavirus 2 (SARS‑CoV‑2) are reduced and delayed in individuals with rheumatic disease, particularly those on B-cell-depleting therapies such as rituximab [3,4]. In December 2021, the US Food and Drug Administration (FDA) issued an emergency use authorization for Paxlovid (nirmatrelvir/ritonavir), an oral antiviral treatment for patients with mild-moderate COVID-19 at high risk for progression to severe disease, and Evusheld (tixagevimab/cilgavimab), a combination of two long-acting monoclonal antibodies given via intramuscular injection, as pre-exposure prophylaxis for patients unable to mount adequate immune responses to vaccination [5,6]. Both agents were expected to remain effective against the variants of concern, including Omicron and its subvariants, during the selected period of interest as per the official CDC health advisory [7]. 

Furthermore, the FDA has continued surveillance for the efficacy of Evusheld and revised the dosing for Evusheld in February 2022 as a higher dose of Evusheld was deemed more likely to prevent infection by the Omicron subvariants BA.1 and BA 1.1. Pharmacokinetic data from June 2022 suggested continued activity against the omicron subvariants BA.2, BA.2.12.1, BA.4, and BA.5 circulating in the US during that time [8]. However, the distribution of antivirals and monoclonal antibodies in early 2022 was hampered due to limited supply and accessibility, especially for Evusheld [9]. 

Gauging public interest through internet searches during the COVID-19 pandemic has been an area of research interest in different specialties. Google Trends and the Baidu index are commonly utilized to research public behavior for COVID-19-related and non-COVID-19-related health topics [10-12]. For example, Kow et al. reported that Google searches for the COVID-19 vaccine surged during the initiation of the vaccination program as well as before the peak vaccination rate in Malaysia [13]. Public interest in Paxlovid and Evusheld through internet searches remains unexplored. Thus, we assessed public interest in Paxlovid and Evusheld using Google Trends at the national and state levels over the first half of 2022, between December 23, 2021, to June 12, 2022.

## Materials and methods

In July 2022, we analyzed Google Trends search queries performed in the US for the period between December 23, 2021, to June 12, 2022, using the following search terms: ''Evusheld'', ''Paxlovid'', ''COVID prophylaxis'' and ''COVID treatment''. We compared Evusheld to Paxlovid, a widely used oral protease inhibitor antiviral medication for the treatment of COVID-19 in patients at high risk for progression to severe COVID-19, including but not limited to patients on corticosteroids or other immunosuppressive medications [14].

Google Trends provides free access to a largely unfiltered sample of actual search requests made to Google and normalizes search data to make comparisons between terms easier. Per Google, each data point is divided by the total searches of the geography and time range it represents to compare relative popularity. The resulting numbers are then scaled on a range of 0 to 100 based on a topic’s proportion to all searches on all topics [15].

We obtained and plotted the relative search volume, ​which represents the normalized search volume in a given period (scale 0-100), for each term. A value of 100 is the peak popularity for the term, and a score of 0 means there was not enough data for the searched term. We used the Centers for Disease Control and Prevention (CDC) data for weekly averages of COVID-19 cases during our period of interest [16]. Additional statistical testing or modeling was not performed as this descriptive study aimed to visually examine patterns in search trends with a focus on the comparison of relative search volumes for different search terms. 

As search trends at a national level may not show variations seen at a state level, we further examined queries at the state level since medication accessibility and public health advisories vary from state to state. We chose Massachusetts and Washington state as representative states, reflecting the authors’ state of residence as well as providing geographical differences.

## Results

For both US- and state-level data, there was a rise in both COVID-19 cases and Paxlovid searches following the discontinuation of the public transportation mask mandate on April 18, 2022 [17]. The number of Evusheld searches remained unchanged throughout our search period at the both US- and state-level analyses. The number of COVID-19 treatment searches increased after April 2022 for the US-level data, although this trend was not demonstrated at the state level.

**Figure 1:**
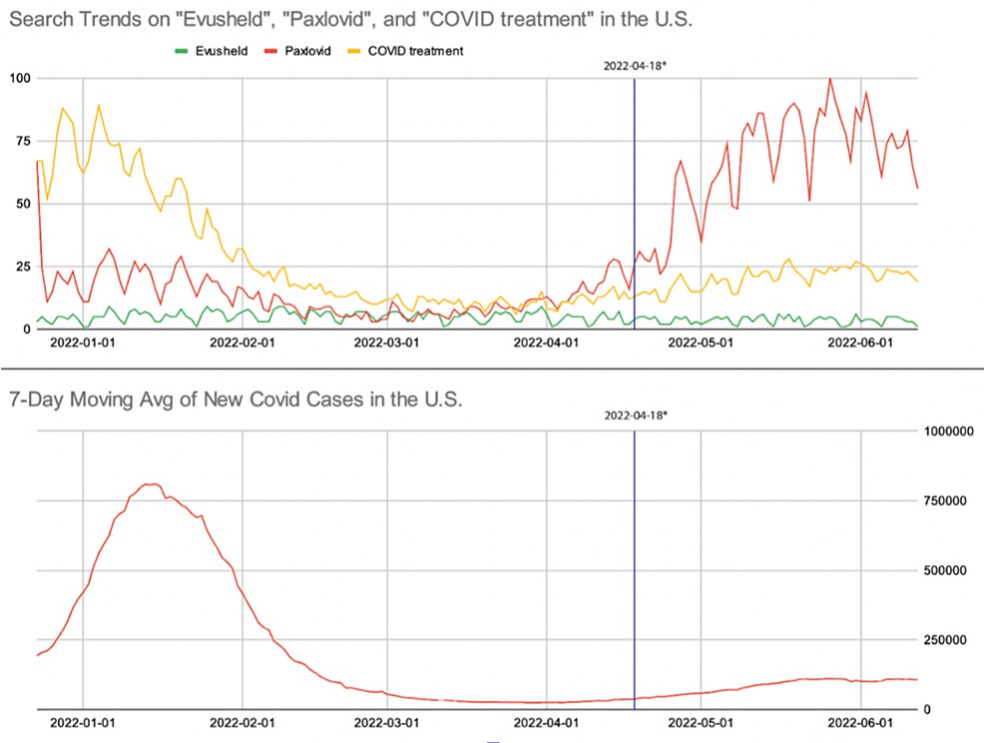
Google Search trends for Evusheld, Paxlovid, and COVID treatment in the United States (top), and weekly averages of new COVID-19 cases in the United States (bottom), from December 23, 2021, to June 12, 2022 Relative search volumes were graphed for Evusheld (green), Paxlovid (red), and COVID treatment (yellow). The blue vertical line represents the date (04-18-2022) that the public transportation mask mandate was lifted in the US. Data was generated on 6-21-2022 via https://trends.google.com/trends/?geo=US

**Figure 2:**
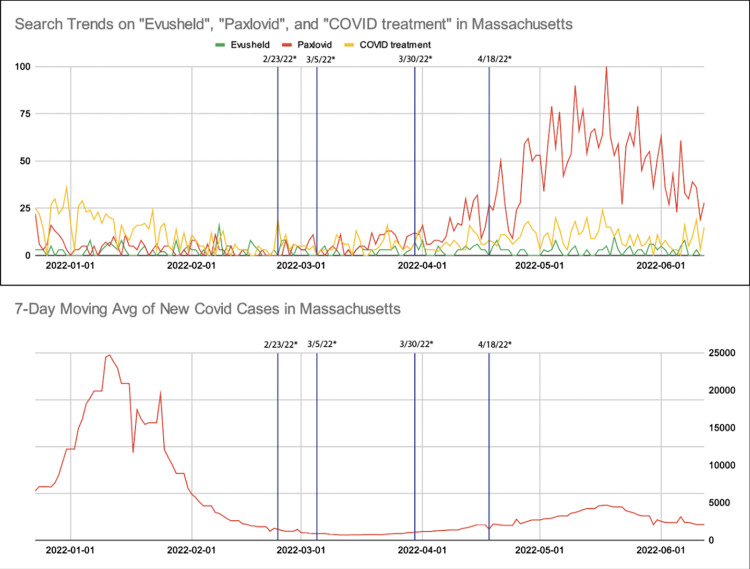
Google Search trends for Evusheld, Paxlovid, and COVID treatment in Massachusetts (top), and weekly averages of new COVID-19 cases in Massachusetts (bottom), from December 23, 2021, to June 12, 2022 Relative search volumes were graphed for Evusheld (green), Paxlovid (red), and COVID treatment (yellow). Blue vertical lines represent the changes in public health advisories in Massachusetts, as detailed below: - 2/23/22: The Massachusetts Department of Public Health reminds Massachusetts residents of the free COVID treatment options for positive high-risk individuals. - 3/5/22: Boston lifts public indoor mask mandate. - 3/30/22: Second booster shot available for immunocompromised individuals and anyone above the age of 50. - 4/18/22: Public transportation mask mandate lifted. Data was generated on 6-21-2022 via https://trends.google.com/trends/?geo=US

These trends for our search terms are listed in line chart format, with the top image showing changes in relative search volume for selected search terms and the bottom image showing the seven-day average of new COVID-19 cases based on the CDC registry (Figures 1, 2). There was a negligible volume of queries for COVID prophylaxis for both the US- and state-level search data; therefore, the results for the search term ''COVID prophylaxis'' are not included in our figures. 

**Figure 3:**
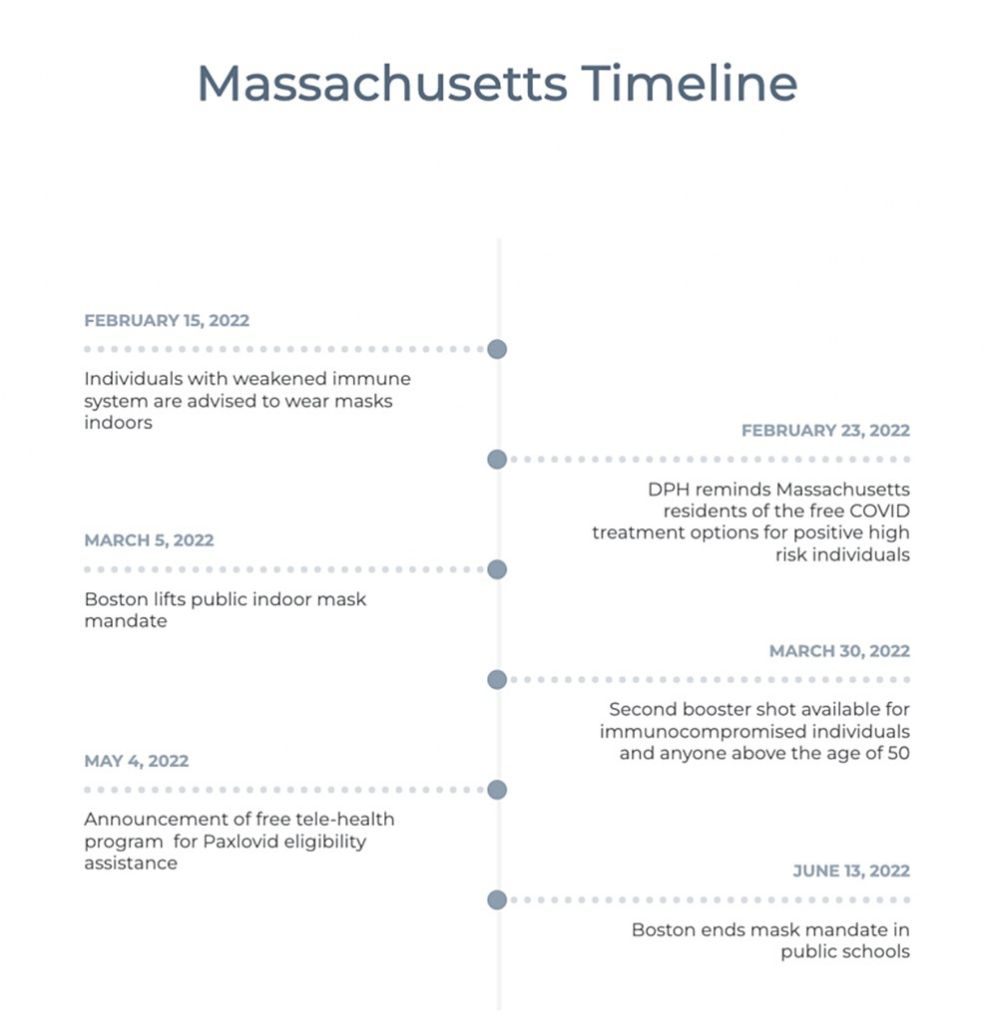
Timeline of public health advisories in the Commonwealth of Massachusetts Data was obtained on 6-21-2022 from https://www.mass.gov and https://www.boston.gov

**Figure 4:**
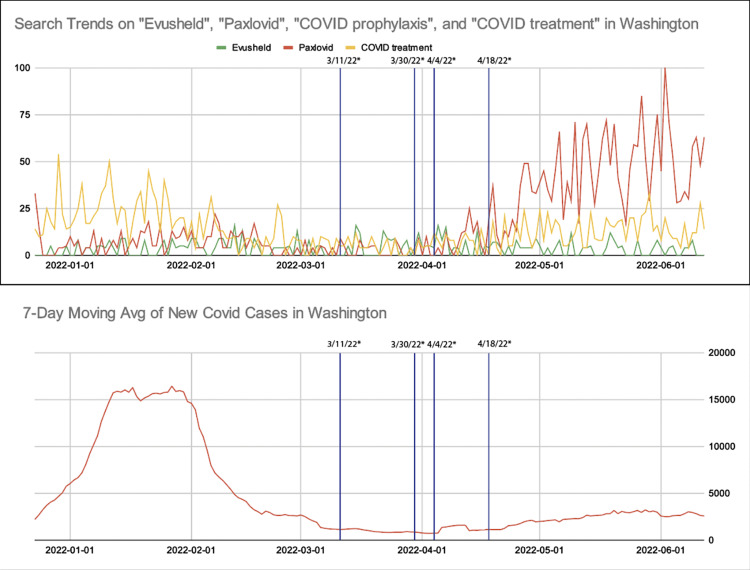
Google Search trends for Evusheld, Paxlovid, and COVID treatment in Washington (top), and weekly averages of new COVID-19 cases in Washington (bottom), from December 23, 2021, to June 12, 2022 Relative search volumes were graphed for Evusheld (green), Paxlovid (red), and COVID treatment (yellow). Blue vertical lines represent the changes in public health advisories in Washington, as detailed below: - 3/11/22: Indoor masking becomes optional. - 3/30/22: Second booster shot recommended for immunocompromised individuals and those who are 50 and older. - 4/4/22: Thousands of doses of Evusheld available in Washington. - 4/18/22: Public transportation mask mandate lifted. Data was generated on 6-21-2022 via https://trends.google.com/trends/?geo=US

**Figure 5:**
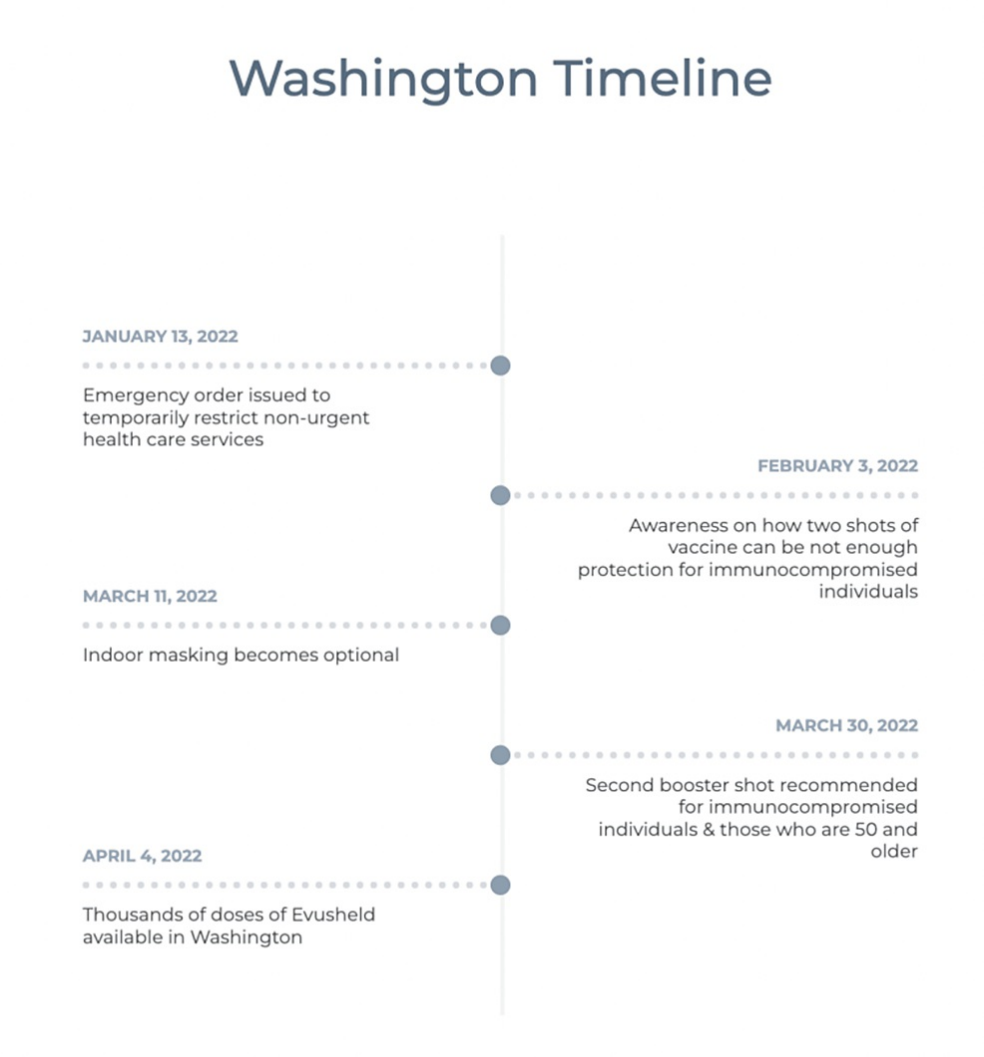
Timeline of public health advisories in the State of Washington Data were obtained on 6-21-2022 from https://wa.gov

Timelines of public health advisories from Massachusetts and Washington are included in Figures 3, 5. Both states had similar public health announcements regarding indoor mask policy in March 2022. This was followed by the termination of the policy on mask mandate while on public transportation, the CDC policy implemented on January 29, 2021, by a federal judge on April 18, 2022 [17]. Figure 4 shows Google Search Trends for Evusheld, Paxlovid, and COVID treatment, along with weekly averages of new COVID-19 cases in Washington. 

## Discussion

We found increasing Google search trends for Paxlovid and COVID-19 treatment over the first half of 2022; however, the same trends were not seen for Evusheld. The plateau in Evusheld searches may reflect low public awareness despite its demonstrated efficacy in reducing the risk of severe COVID-19 in immunocompromised individuals without an adequately protective response to available vaccines [18]. Further efforts are needed to increase public awareness of adjunctive prophylactic measures such as Evusheld for those who are at high risk for severe outcomes of COVID-19 infection.

Multiple real-world studies have demonstrated the efficacy of Evusheld (tixagevimab/cilgavimab), a monoclonal antibody combination with long-acting protection against severe COVID-19 outcomes. A population-based study from Israel using data from December 2021 to April 2022 (during the initial period of omicron variant dominance) found lower odds of infection among those who received Evusheld versus those who did not [19]. In a French cohort of individuals with immune-mediated inflammatory diseases on immunosuppressive therapy who had received four COVID-19 vaccine doses (n=70), 17 had inadequate humoral responses [18]. Of these 17, 10 received Evusheld as pre-exposure prophylaxis, and only one had a documented breakthrough infection during the omicron wave, compared to seven who had not received Evusheld. Boekel et al. recently reported that patients who are on anti-CD20 therapy (e.g., rituximab) remain at higher risk for COVID-19-related hospitalization with omicron variants [20]. Finally, a descriptive retrospective study from the Cleveland Clinic studied outcomes for immunocompromised individuals (including immune-mediated inflammatory diseases on B-cell depleting therapies) who received Evusheld (n=412), and it showed that the vast majority who did have breakthrough COVID-19 infection had a non-severe clinical course (11 of 12) [21].

To our knowledge, no prior studies have assessed public interest in Evusheld or other COVID-19 therapeutics for high-risk individuals. This remains critical given the data showing immunocompromised patients are likely to remain more vulnerable to severe COVID-19 outcomes even after vaccination [9]. An editorial called for the improved education of patients and clinicians amidst changing guidelines and available treatment strategies [9]. In addition, it is important to ensure methods for more equitable distribution at both federal and state levels. A US study showed that despite an increase in the number of dispensed antivirals, dispensing rates were lowest in high-vulnerability neighborhoods despite having more dispensing sites [22]. Improving public awareness would also help ensure more equitable access to antiviral strategies.

Our findings suggest that, alongside improving access and supply, there should be a focus on identifying other barriers to administration, including improving awareness for both patients and clinicians of the available therapies for those who remain at high risk despite vaccination. In this regard, Evusheld remains the only pre-exposure prophylaxis (PrEP) for COVID-19 and was authorized to be administered every six months. An FDA press release issued on October 3^rd^, 2022, continues to recommend Evusheld as an appropriate option for PrEP to prevent severe COVID-19, in combination with other preventative measures like getting vaccinated and boosted as recommended, as Evusheld still offers protection against many of the currently circulating variants and may offer protection against future variants [8]. 

Our study has several limitations to note. First, using Google Trends, we graphed the relative search volume and not the absolute number of searches which might have prevented us from accurately capturing trends in Evusheld searches. Second, there may be a potential bias arising from data capture from one search engine. Finally, it was not possible to extrapolate public interest data to medication receipt or administration. 

Strengths of this descriptive study include the use of dynamic and readily accessible data to gauge public interest on a national scale. The similarity of trends between the US- and state-level data support the generalizability of the data. Other strengths of our study include the capture of six months of data following the FDA emergency use authorizations of both Evusheld and Paxlovid, as well as the use of Paxlovid as a comparator to Evusheld.

## Conclusions

To the best of our knowledge, this is the first study assessing public interest in COVID-19 therapeutic agents for high-risk individuals, Paxlovid and Evusheld, by utilizing Google Trends, a freely available tool to monitor online searching behavior. Our Google Trends analysis demonstrates a plateau in the public interest for Evusheld at both the national and state levels despite increasing interest in other COVID-19 therapeutics like Paxlovid. This may reflect low public awareness of Evusheld, but further studies using different platforms would be helpful to assess its replicability. Nevertheless, given its protective benefit against severe COVID-19 outcomes in our most vulnerable immunocompromised populations, continued education through public health initiatives should be undertaken. Efforts to increase awareness for Evusheld, such as dissemination of infographics in clinics and online, as well as other additional prevention and risk mitigation strategies, will continue to have a high impact on the most vulnerable high-risk individuals, including immunocompromised patients.
